# Effect of WIN55-212-2 and Consequences of Extinction Training on Conditioned Fear Memory in PTSD Male Rats

**DOI:** 10.29252/NIRP.BCN.8.6.493

**Published:** 2017

**Authors:** Malihe Ghasemi, Kataneh Abrari, Iran Goudarzi, Ali Rashidy-Pour

**Affiliations:** 1. Department of Physiology, School of Biology, Damghan University, Damghan, Iran.; 2. Physiology Research Center, Semnan University of Medical Sciences, Semnan, Iran.

**Keywords:** Post-traumatic stress disorder, Cannabinoids, Win 55212-2, Extinction

## Abstract

**Introduction:**

This study investigates the effects of cannabinoid agonist WIN55-212-2 on acquisition and consolidation phases of the fear memory extinction and also on anxiety and motor activity.

**Methods:**

In this study, we used SPS & S model to induce post-traumatic stress disorder. One week after SPS, to establish a conditioned fear memory, rats received an electric foot shock within shock chamber. After 24 h, for extinction training, the rats were placed back to the chamber for 9 min, without receiving any shock. In 3 consecutive days and on days 17, 24 and 37, extinction tests were carried out and the freezing behavior was evaluated. Thirty minutes before the first three extinction tests, animals received IP injections of WIN or vehicle. Anxiety-like behavior examined with elevated plus-maze and motor activity with open field, 32 days after conditioning.

**Results:**

Exaggerated and continued conditioned fear memory observed in SPS & S group compared with shock group. IP injection of a 0.25 mg/kg dose of WIN before extinction training led to reducing fear responses in animals.

**Conclusion:**

IP injection of WIN increased acquisition or consolidation of fear memory extinction. SPS & S caused anxiety and this effect improved by the agonist (0.25 mg/kg).

## 1. Introduction

Post-Traumatic Stress Disorder (PTSD) is a stress-related mental disorder characterized by four symptoms: avoidance behavior, re-experiencing, emotional numbing, and hyperarousal resulting from an emotionally traumatic event with noted threat (Pitman et al., 2012).

The disorder can be considered a maladaptation to traumatic stressors, with altered fear-related learning (fear conditioning) and extinction, behavioral sensitization, and alterations in brain areas function and neurotransmitter systems closely link to these processes (Schafer, Christenson, Teague, & Grifo, 1973).

Memory damage is a prominent feature of PTSD. Clinical studies of patients with PTSD showed that these patients have two major problems related to memory: re-experiencing traumatic events and avoidance of stimuli that caused by trauma. Patients often recall the memories of the traumatic events and cannot forget it. The main reason for memory disruption is the failure of fear extinction (Milad et al., 2009).

Humans and animals bring up fear of signs or cues paired with annoying events. Fear conditioning is a kind of learning, association between Conditioned Stimulus (CS) and a harmful Unconditioned Stimulus (US). In rodent models, usually the CS is a chamber and the US is foot shock. Fear extinction is a new learning, a negative association between CS and US (Myers & Davis, 2006; Orsini & Maren, 2012). In this new learning, the fear conditioning memory is intact (Myers & Davis, 2002; 2006). So, the brain stores opposite memory about the same CS.

Although the mechanism of neuronal circuitry of fear extinction is not entirely known, some studies have suggested the three structures of hippocampus, amygdala, and prefrontal cortex are involved in memory extinction. Previous studies showed that CS stores in hippocampal– cortical networks, while cue and unconditioned stimulus are processed in the Basolateral Amygdala (BLA). BLA is critical for the production of fear conditioning during extinction process, the medial prefrontal cortex joins the hippocampal-amygdala circuit and by inhibiting the amygdale, decrease the fear response (Bouton, 2004; Myers & Davis, 2006; Orsini & Maren, 2012). As a kind of learning, extinction has three phases: acquisition, consolidation, and retrieval. Acquisition of extinction takes place when conditioned responses are decreasing within an extinction training process. Consolidation phase is stabilization of a long-term memory for extinction. Then, representation of the CS recalls the extinction memory, called retrieval (Sotres-Bayon, Cain, & LeDoux, 2006).

MRI studies showed structural and functional changes in these areas in patients with PTSD. Previous studies suggested that damage to neuronal connections between these structures leads to disturbance in memory extinction in patients with PTSD (Koenigs & Grafman, 2009).

Physiological and behavioral changes observed in animals exposed to Single Prolonged Stress (SPS) could fitly represent pathophysiological process and core symptomatology of PTSD, including anxiety behavior and cognitive impairments. SPS paradigms have been extensively applied in the investigation of PTSD. Previous studies have further shown that unavoidable electric foot Shock (S) added to formal SPS procedures significantly enhanced conditioned and sensitized fear responses.

Based on studies of the neural circuits involved in fear memory extinction, several major neurotransmitters are involved in conditioned fear extinction: Gamma Amino Butyric Acid (GABA), glutamate, acetylcholine, dopamine and opioids and endocannabinoids (Chambers et al., 1999; Heim & Nemeroff, 2009; Vaiva et al., 2004).

Cannabinoids do their effects through CB1 and CB2 receptors. CB1 receptors are expressed in large amounts in the central nervous system parts, including spinal cord, brain stem, cerebral cortex, basal ganglia, amygdala and hippocampus, whereas CB2 receptors are mainly found in the peripheral tissues such as immune cells, liver, spleen, and testicles (Svizenska, Dubovy, & Sulcova, 2008).

Previous studies have shown that the endocannabinoid system are involved in learning and memory processes by acting on hippocampus, amygdala, and cerebral cortex (Moreira & Lutz, 2008). Also, studies have confirmed the role of this system in the modulation of anxiety. One evidence of endocannabinoid system’s intervention in emotional learning and anxiety is huge presence of the CB1 receptors and also the presence of endocannabinoids in regions that are important for anxiety and emotion, including the amygdala and hippocampus (Iversen, 2003; Maccarrone, 2005).

A great number of studies suggest an important role of the cannabinoid system in controlling the fear memory extinction. Pharmacological blockade of the CB1 cannabinoid receptor reduces extinction of fear conditioning and spatial memory in rodents (Chhatwal, Davis, Maguschak, & Ressler, 2004; Lafenetre, Chaouloff, & Marsicano, 2007).

As several human psychiatric disorders such as PTSD damage adaptation to changing environmental conditions, the hope is rising that the endocannabinoid system might be a valuable therapeutic target to treat this disorder. We decided to find out the effect of the cannabinoid receptor agonist WIN 55,212-2, on fear conditioned memory extinction and PTSD impelled anxiety in male rats.

## 2. Methods

### 2.1. Animals

Wistar rats (180–200 g) were kept at cages (five in each cage), in 12-h light/dark cycle, and fed and watered ad libitum. All processes were conducted according to the National Institutes of Health Guide for care and use of laboratory animals. Every attempt was made to downgrade the number of animals used per group and to reduce animals used throughout all experimental procedures.

### 2.2. Enhanced single prolonged stress procedure

**Figure 1.**
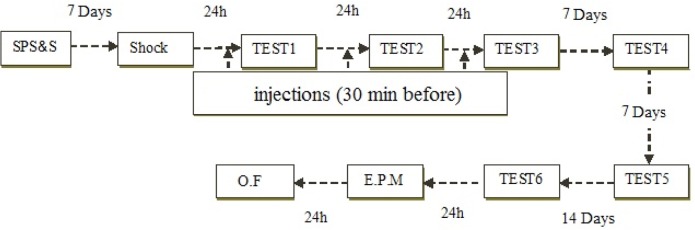
Timeline of experiments (Experimental groups described in section 2.7)

Detailed SPS procedure has been described in previous studies (Wang et al., 2008; Yamamoto et al., 2009). Rats maintained for 2 h in restrainer, immediately forced swimming for 20 min in 24°C water contained in an acrylic cylinder (24×50 cm). After 15 min of recovery, animals were subjected to diethyl ether until they lost astuteness. When they recovered (about 30 min), a single electric foot shock (1 s, 1.5 mA) delivered via metal grids installed in the bottom of the chamber (Section 2.3). Stressed rats remained in the shock chamber for another 60 s, before returning to the home cages (Figure 1).

### 2.3. Shock application in fear conditioning apparatus

Fear conditioning system (TSE, Bad Homburg, Germany) was used to study contextual fear conditioning of each rat. Contextual fear conditioning took place in a chamber. The walls of the box were made of clear Plexiglas. The box contains a loud speaker and light bulb. The floor of the box contains 28 stainless steel bars (6 mm in diameter, 12 mm apart) through which foot shock could apply. The chamber became clear by a single light, and cleaned before and after use. A software program was used to check the box and to collect and store all experimental data for analysis.

One week after SPS, stressed rats received the electrical foot shock within the fear conditioning chamber. After 180 s, they received one shock (1.5 mA) for 1 s. Stressed rats held in the shock chamber for another 60 s before returning to the home cages (as shown in section 2.2).

### 2.4. Extinction test of conditioned fear memory

Twenty-four hours after contextual fear conditioning, the animals were exposed to the conditioning chamber for 9 min and their freezing behavior was evaluated. “Freezing” behavior is defined as immobility except for respiration movements. Fear is quantified as the time (in seconds) spent “freezing”. This extinction procedure executed three times at 24-h intervals. Remote memory were tested by three tests, two of which conducted one week after test 3 with one week interval (tests 4 and 5) and the final one (test 6) was conducted one month after test 3. However, the percentage of freezing during the first extinction session was used to examine any possible within-session effects of drug treatment. The animals were treated with WIN (0.25, 1.25 mg/kg, IP) or vehicle, half an hour before the first three extinction tests (as defined in section 2.2).

### 2.5. Open field test

In the open field test, each rat was placed at the center of a cubic chamber (40×40×30 cm). All rats were habituated for 20 min before starting the experiment. Test lasted for 5 min. The room was illuminated with indirect red lighting. The floor of the box was divided into 9 equal squares (3×3 cm). The number of crossings was counted. Data were analyzed by using Ethovision ver.5. “Center” was defined by the number of entries into the central squares divided by the total squares crossing.

### 2.6. Elevated plus maze test

Animals were placed in a wooden elevated plus maze consisting of two opposite open arms (50×10 cm), two opposite closed arms (50×10×40 cm), and a central area measuring 10×10 cm. The plus maze was mounted on a base, elevated 50 cm above the floor. Animals were placed at the central area, facing the open arms. In a 5-minute test, the mouse’s entering to the open/closed arms is counted and recorded, and also the time spent on each arm. Two main factors to compare were as follows: “OAE=number of ingresses into the open arms/(number of entries onto the open arms+closed arms)” and “OAT=time stay in the open arms/(time spent in the open arms+closed arms).”

### 2.7. Experimental groups

Rats were randomly assigned to SPS or shock groups (9–10 animals in each group). SPS group: rats of this group were treated through the procedures described in sections 2.2 and 2.3. Shock group: without experiencing SPS procedure, these rats received a shock that conducted through the procedure described in section 2.3.

### 2.8. Drugs and treatment

WIN 55,212–2 (Tocris, USA) first dissolved in 100% DMSO and subsequently was diluted in phosphate buffer to reach a final DMSO concentration of 2%. The control solution consisted of a drug vehicle. All drug doses, selected according to previous reports, were given intraperitoneally in a volume of 0.2 mL/100 g of body weight. WIN or vehicle was administered 30 min before behavioral tests 1 to 3.

### 2.9. Statistical analysis

Data were presented as mean±SEM and analyzed by 1- and 2-way ANOVA for repeated measurements with SPSS 16.0. Tukey post hoc test was performed to find out the source of the detected significant differences. P<0.05 was considered significant.

## 3. Results

### 3.1. Experiment 1: Effects of WIN 55,212–2 on contextual fear memory extinction

The goal of this experiment was to study the effects of WIN (0.25, 1.25 mg/kg, IP) on fear conditioned extinction. So, one day after conditioning, the animals subjected to the conditioning chamber for 9 min and the freezing behavior measured. This extinction procedure performed three times at 24-h intervals (tests 1 to 3). Rats were randomly divided into the groups described in section 2.6. The animals were treated with WIN or control solution before each extinction session.

**Figure 2.**
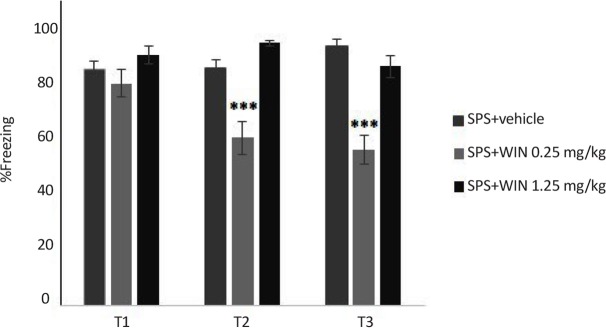
Administration of WIN produced a dose dependent effect on the extinction process *** P<0.001 as compared with SPS+vehicle group. Rats injected with WIN (0.25 mg/kg) prior to test reached a lower level of freezing compared to rat injected with vehicle. Data were presented as Mean±SEM.

**Figure 3.**
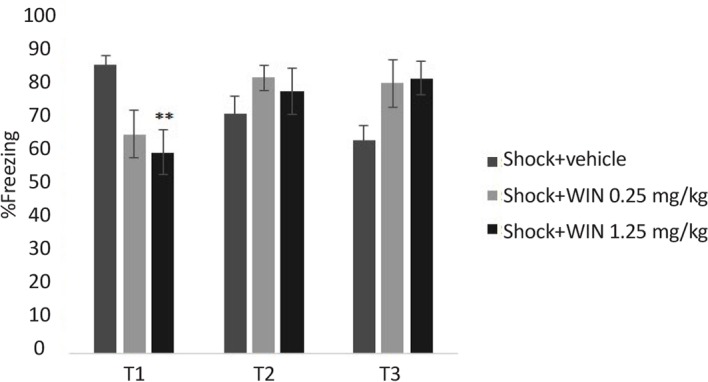
Effects of WIN on shock groups Freezing behavior in shock +WIN (1.25 mg/kg) group, only in the first test, was lower than shock+vehicle group. **P<0.01 as compared with shock+vehicle group. Data were presented as Mean±SEM.

The effects of WIN treatment on contextual fear memory are given in Figures 2 and 3. Two-way ANOVA with repeated measures revealed no significant effects of groups but significant effects of days and “day×group” interaction (F_10,100_=17.507, P<0.01).

Post hoc comparisons pointed out that the percentage of freezing in SPS+vehicle group was significantly higher than shock+vehicle group in tests 2 and 3.

Administration of WIN promoted a dose dependent effect on the extinction. The lowest levels of freezing appeared to occur in rats injected with WIN (0.25 mg/kg) before testing. An examination of the freezing data over the second and third extinction sessions revealed that rats injected with WIN (0.25 mg/kg) prior to test reached a lower level of freezing compared to rat injected with vehicle (P<0.001) (Figure 2).

There was significant difference between the shock +1.25 WIN group and shock+vehicle group (P<0.01). This difference was only seen in the first, but not the second or third tests (Figure 3). These results may indicate that WIN at with high dose temporarily weakens acquisition.

### 3.2. Experiment 2: Stability of the cannabinoid agonist WIN effects on contextual fear memory extinction

**Figure 4.**
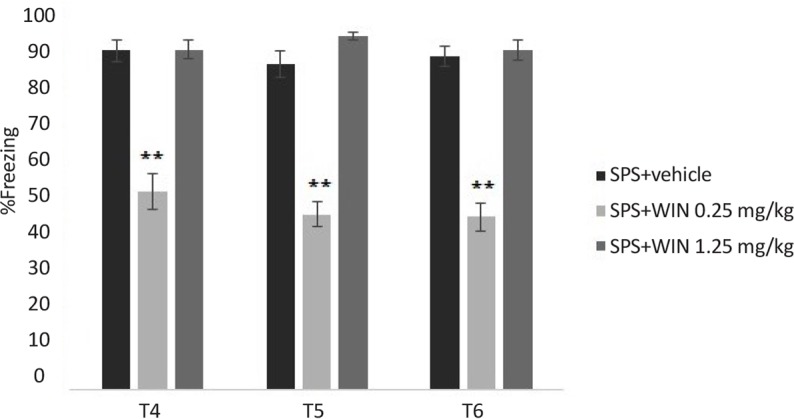
Stability of the cannabinoid agonist WIN effects on contextual fear memory extinction in SPS groups. Administering WIN (0.25 mg/kg) significantly decreased the freezing time in SPS +0.25WIN group, compared to SPS+vehicle group in all three tests. **P<0.01 as compared with SPS+vehicle group. Data were presented as Mean±SEM.

One (test 4), two (test 5) and three (test 6) weeks after the third test, the animals were re-exposed to the conditioning chamber for 9 min and the freezing behavior was evaluated. The longer effect of WIN (0.25 mg/kg) on extinction of contextual fear memory in rats is shown in Figure 4. Two-way ANOVA revealed no significant effect of days (F_2,100_=0.997, P=0.323) but significant effects of groups (F_5,50_=15.011, P<0.000), and “day×group” interaction (F_10,100_=2.386, P<0.05) (Figure 4).

Post hoc comparisons showed administering WIN (0.25 mg/kg) significantly decreased the freezing time in SPS+WIN (0.25 mg/kg) group, compared to SPS+vehicle group in all three tests (P<0.01) (Figure 4), suggesting a constant facilitative effect of WIN on the extinction of contextual fear memory.

There were no significant difference between shock+WIN treated groups and shock+vehicle group in all tests. These results may suggest that WIN had no permanent effect on extinction in conditioned groups.

### 3.3. Experiment 3: Within group comparison of contextual fear memory extinction

Animals in each of the experimental groups underwent 6 extinction tests and half an hour before the first three extinction tests they received vehicle or WIN, and the three following tests accomplished without any injections. To evaluate the stability of the effects of treatment, we compared the within group freezing behavior of the animals in test one and test 6, by paired t test.

In SPS+vehicle group, there was no significant difference in freezing behavior between test 1 and 6. In other words, 54 minute exposure to condition chamber could not lead to the extinction of fear memory in this group. This result was the same as that SPS+WIN (1.25 mg/kg) group.

In SPS+WIN (0.25 mg/kg) group, significant decrease was observed in final test as compared to the first one, suggesting a facilitative effect of this dose in the extinction of contextual fear conditioning.

**Figure 5.**
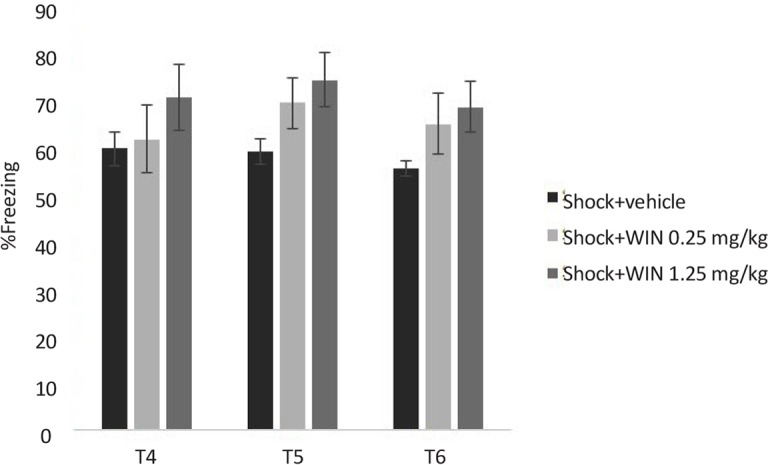
Stability of the cannabinoid agonist WIN effects on contextual fear memory extinction in shock groups There were no significant deference between shock +WIN treated groups and shock+vehicle group in all tests. Data were presented as Mean±SEM.

Also a significant decrease was observed in shock+vehicle group from test 1 to 6, suggesting a weakening of conditioned fear response. In contrast to this group, a significant increase was observed in shock+WIN (1.25 mg/kg) group, so the higher dose of WIN (1.25 mg/kg) disrupted the extinction of conditioned fear as shown by the lack of reduction in the freezing time across the trials (Figure 5).

Administering WIN promoted a dose dependent effect on the extinction. The shock group treated with the lowest dose of WIN (shock+0.25 WIN group) presented no difference in freezing time during 54 min exposure. When a low dose of WIN (0.25 mg/kg) was used, freezing in later sessions decreased, consistent with the idea the acquisition or consolidation of the extinction memory may have increased.

Experiment 3 explained that injections of 1.25 mg/kg WIN or vehicle were largely without effect on extinction. In other words, freezing remained high when repeated extinction sessions accomplished in SPS+vehicle and SPS+WIN (1.25 mg/kg) groups, consistent with the idea that acquisition or consolidation of the extinction memory was weakened by these treatments. Multiple mechanisms may contribute to the effects of ethanol on the development and expression of extinction.

### 3.4. Experiment 4: Effects of the cannabinoid agonist WIN on anxiety like behavior

To find out the effect of cannabinoid agonist WIN on anxiety, animals were tested for anxiety behavior, using the EPM at the end of the experiments. As noted in section 2.5.2, two main factors of OAE and OAT were evaluated in all experimental groups as indicators of anxiety. One-way ANOVA revealed a significant difference between groups on the time spent exploring in the open arms (OAT) (F_5,50_=44.391, P<0.001) and the count number of entries to the open arms (F_5,50_=38.37, P<0.001).

**Figure 6.**
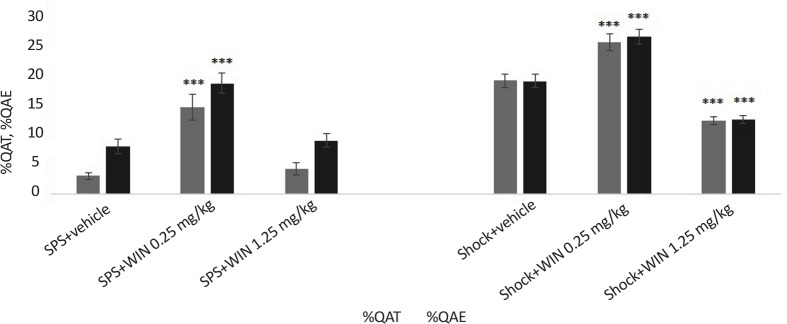
Effects of the cannabinoid agonist WIN on anxiety-like behavior *** P<0.001 as compared with the same factor in related control group. Data were presented as Mean±SEM.

Post hoc Tuckey comparison suggested the SPS group treated with WIN (0.25 mg/kg) showed less anxiety-like behavior, spent more time (P=0.071) and performing more entries in the open arms (P=0.030) compared with SPS+vehicle group (Figure 6). Also, the same effect of Win (0.25 mg/kg) was observed in shock+WIN (0.25 mg/kg) group compared to shock+vehicle group. Percentage values of OAE and OAT in shock+vehicle group were significantly more than in SPS+vehicle group (Figure 6).

### 3.5. Experiment 5: Effects of the cannabinoid agonist WIN on motor activity

The effects of WIN (0.25 or 1.25 mg/kg) on the main factor (number of crossing) in the open field test and also the motor activity of animals have been measured. Oneway ANOVA revealed no significant effect of treatment on the number of squares crossed (F_5,50_=1.81, P=0.12).

## 4. Discussion

### 4.1. Contextual fear memory extinction

Findings showed that after one week, conditioned response increased in the PTSD rats (SPS+vehicle group) as compared to shock+vehicle rats. This improvement lasted even after three weeks. This outcome was predictable because impairment of memory extinction is one of the symptoms of PTSD.

In PTSD rats, acute systemic administration of CB1 agonist (WIN 55,212-2) before extinction tests, have shown to ease fear extinction. Our results suggest that the extinction of contextual fear memory in PTSD rats may be eased by the cannabinoid agonist WIN. Low dose of the cannabinoid agonist WIN (0.25 mg/kg) facilitated the extinction of conditioned fear. This result is consistent with Parker et al. (2004) findings indicating that low dose of Δ9-tetrahydrocannabinol and cannabidiol promote extinction of conditioned place preference in rats (Parker, Burton, Sorge, Yakiwchuk, & Mechoulam, 2004).

Since the injections of agonist were accomplished 30 min before extinction tests and half-life of the drug was about 2 hours, this decrease may be because of the effect of treatment on all three phases of extinction, i.e. strengthening the acquisition, weakening the retrieval, or consolidation. Detailed evaluation of the agonist effects needs further investigation.

This effect may occur through neurotransmitter systems. CB1 receptors are presynaptic receptors. They reduce the neurotransmitters release such as GABA, glutamate, dopamine, acetylcholine, and so on (E Alger, 2002). CB1 receptors were densely localized within brain structures that are critical for learning and retrieval of extinction memories (ventromedial, prefrontal cortex and hippocampus) (Pamplona & Takahashi, 2006).

Results showed that WIN 55,212-2 doses had no effect on fear extinction in shock group, as revealed in lack of freezing difference between shock+vehicle and shock+WIN groups. There are conflicting evidence that administration of cannabinoid agonists and their reup-take inhibitors before extinction training affects the acquisition and retrieval of memory extinction (Kamprath et al., 2006; Lin, 2006). Some of the previous studies suggested that cannabinoids may produce a state-dependent learning by affecting adrenergic receptors.

### 4.2. Stability of WIN 55,212-2 effect on contextual fear memory extinction:

As described in section 2.2, three extinction tests were accomplished after test 3, without prior injections. The aim was to examine the maintenance of agonist’s effect. Animals who received lower dose of WIN 55,212-2 showed less freezing as compared with SPS+vehicle group in all tests from test 2 to 6. The effect of agonist remained till test 6, which clearly highlights the long-term facilitative effects of WIN on the extinction of conditioned fear.

Our results suggest the extinction of contextual fear memory in rats may be facilitated by the cannabinoid agonist WIN with long lasting effects. Within group comparison of freezing behavior showed the strengthened extinction in animals was treated with 0.25 mg/kg WIN. Decrease in the percentage of freezing from test 1 to 6 in SPS+WIN (0.25 mg/kg) group revealed a positive effect of WIN on memory extinction, the effect that remains for a month after final injection. This consistent result as supported by previous results showed the facilitative effect of low dose of specific and nonspecific cannabinoid receptor agonist on extinction of conditioned fear memory (Pamplona & Takahashi, 2006).

Similar to the previous reports, repeating the extinction tests may gradually reduce fear responses (Quirk et al., 2010); this is what we saw in the shock+vehicle group, i.e. amount of freezing decreased from test 1 to 6. Administration of WIN (0.25 mg/kg) in shock+WIN (0.25 mg/kg) group prevented the effects of the extinction tests, as revealed in high levels of freezing in test 6. When animals in shock group treated with high dose of WIN (1.25 mg/kg), freezing in later tests increased, consistent with the idea that to consolidation of extinction memory may be disrupted. Together, these findings suggest that multiple mechanisms may contribute to the effects of WIN on expressing extinction in conditioned rat in shock group and PTSD rats in SPS group.

Since the traumatic events lead to destroy many regions of the brain, particularly the hippocampus that associated with memory and because of one of the fundamental problems in patients with PTSD is inability of forgetting the traumatic memory, using cannabinoid receptor agonist (WIN 55,212-2) in a dose-dependent manner, after traumatic events, may be useful in treating memory disorders. A drug which helps extinction of conditioned fear in laboratory animals may also utilized with success in humans (Walker & Davis, 2002), so pharmacotherapies directed at the cannabinoid system may represent a viable approach to treat a variety of psychiatric disorders related to the retrieval of fear memories, such as panic, phobias, and PTSD.

### 4.3. Anxiety like behavior

Elevated plus maze is a popular model to assess anxiety in rodents (Walf & Frye, 2007). Two factors, percentage of time spent in the open arms and the number of entering to open arms were evaluated as indexes of anxiety like behavior. Low dose of CB1 receptor agonist (WIN 55,212-2) had anti-anxiety effects on PTSD animals. Both factors significantly increased in the WIN treated group as compared with the control group. Increased percentage of these factors showed reduced anxiety levels.

Several studies confirm our results about anxiolytic effects of cannabinoid receptor agonists. For example, previous reports showed that low doses of CP55940 and tetrahydrocannabinol such as CB1 receptor agonist, had anxiolytic effects in different animal models for anxiety.

The study also showed that the effect is dose-dependent. Low dose of WIN 55,212-2 (0.25 mg/kg) has anxiolytic and high-dose has (1.25 mg/kg) anxiogenic effects. This result is consistent with other findings that high doses of cannabinoid agonists such as HU-210 and CP55940 have anxiogenic effects. Cannabinoid anxiogenic effect is one of the reasons for discontinuation of cannabinoid usage in human studies. Interestingly, we showed anxiolytic effect of WIN 55,212-2 using low dose and anxiogenic effect using higher dose in shock groups. Our findings are consistent with other studies results (Haller, Bakos, Szirmay, Ledent, & Freund, 2002; Patel & Hillard, 2006; Rubino et al., 2008).

Although we did not evaluate the mechanism of WIN effects on anxiety but its modulatory effect may occur via some neurotransmitter systems, for example cholinergic system. There was a modulatory effect of acetylcholine on anxiety (Tzavara, Wade, & Nomikos, 2003), and some evidence suggested that using low and high dose of WIN, respectively elicits and inhibits the long term secretion of acetylcholine in hippocampal circuits (Degroot & Treit, 2002). The modulatory effects of WIN on anxiety in plus-maze of our study may have been conducted via hippocampal cholinergic systems. Usage of cannabinoid receptor agonist (WIN 55,212-2) after traumatic events could, dose dependently, reduce the level of anxiety in PTSD patients.

### 4.4. Motor activity

Behavioral tests, such as the open-field task, allow to evaluate motor activity, by measuring the number of squares crossed. Previous studies have shown no motor deficits in PTSD patients (Cukor, Spitalnick, Difede, Rizzo, & Rothbaum, 2009). The present study found out no difference in motor activity of SPS+vehicle and shock+vehicle groups. This result confirms that PTSD animals are not suffering from damaged motor activity. Also, there was no difference between WIN treated groups and SPS+vehicle group. This finding matches with other results that show WIN or tetrahydrocannabinol does not affect motor activity (Corbille et al., 2007).

In conclusion, although the underlying mechanisms remain to be determined, the present results provide evidence for the existence of a facilitative effect of CB1 agonist (WIN 55,212-2, 0.25 mg/kg) on the extinction of conditioned fear. This effect is a long lasting effect.

This study also showed that WIN 55,212-2 may be useful in treating anxiety, in a dose-dependent manner, after traumatic events. Low dose of WIN 55,212-2 (0.25 mg/kg) has anxiolytic and high-dose (1.25 mg/kg) has anxiogenic effects.
